# Ictal fear of death with preserved context dependent speech and postictal amnesia in focal epilepsy

**DOI:** 10.3389/fneur.2026.1784636

**Published:** 2026-03-04

**Authors:** Christopher Saouda, Yasmeen Kassem-Scott, Elham El Hallak, Prarthana Hareesh, Victoria Vinarsky, Alexandra Eid, Yamane Makke, Mohamad Zakaria Koubeissi

**Affiliations:** 1 Department of Neurology and Rehabilitation Medicine, George Washington University, Washington, DC, United States; 2 Department of Neuroscience and Behavior, Vassar College, Poughkeepsie, NY, United States; 3 Harvard Center for Brain Science, Harvard University, Cambridge, MA, United States; 4 Department of Neurology, Wellspan Health, Chambersburg, PA, United States

**Keywords:** amnesia, death, fear, focal, language

## Abstract

**Introduction:**

Seizure semiology reflects dynamic interactions among ictal activity, internal state, and environmental context, and often involves distributed neural networks beyond the epileptogenic focus.

**Methods:**

We report a 27-year-old right-handed, blind, bilingual man with focal epilepsy whose seizures were characterized by intense fear of imminent death, loud and coherent vocalizations, context-dependent bilingual language use, accurate autobiographical references, preserved responsiveness, and complete postictal amnesia.

**Results:**

During seizures, the patient consistently addressed his mother in Urdu or English while speaking exclusively in English to medical staff, demonstrating preserved pragmatic awareness; nevertheless, he had no recollection of events afterward and expressed surprise when he heard his ictal vocalizations on the recorded video. Brain MRI revealed a left middle cranial fossa arachnoid cyst abutting the medial temporal lobe and ictal onset was in the right anterior temporal region.

**Discussion:**

This case expands the spectrum of ictal fear and ictal speech by illustrating the convergence of multilingual communication, autobiographical integration, and pure postictal amnesia, highlighting the engagement of distributed limbic, interoceptive, and medial frontal networks.

## Highlights

- During seizures, the patient consistently spoke Urdu/English to his mother but English only to staff, suggesting preserved pragmatic social awareness despite ongoing ictal activity.
- Seizures featured accurate personal references (including to a deceased sibling) and intense fear of imminent death, followed by total amnesia for the events.

## Introduction

Seizure semiology reflects dynamic interactions between the ictal discharge, the physical and mental state of the individual, and the surrounding environmental context. These interactions, conceptualized within cognitive neuroscience through the framework of embodiment, introduce important methodological challenges for the analysis and classification of seizure phenomena (1).

Although focal seizures in each patient are often stereotyped, seizure expression may vary substantially depending on internal state and external circumstances, even when certain ictal signs remain consistent, such as automatisms.

Importantly, many seizure manifestations result from the engagement of widely distributed neural networks, despite originating from a spatially limited epileptogenic focus. Complex seizure semiologies incorporating emotional behavior, social interaction, and language production cannot be adequately explained by activation of a restricted cortical region alone; instead, a network-level perspective is essential to account for their phenomenology.

We report the case of a 27-year-old right-handed man with focal epilepsy whose seizures are characterized by loud, fearful vocalizations containing linguistically appropriate and biographically accurate speech. During seizures, he addresses his bilingual mother in either English or Urdu, while speaking exclusively in English to staff. Responsiveness is preserved during events, followed by complete postictal amnesia. This case illustrates the context-dependent engagement of language and emotional networks during focal seizures and highlights the role of embodied and socially embedded processes in seizure semiology.

## Case report

The patient is a 27-year-old right-handed bilingual man with equal proficiency in Urdu and English. He has a history of congenital cataracts resulting in blindness, who developed epilepsy at 16 years of age. There was no family history of epilepsy. His sister had congenital blindness and intellectual disability and died from an infection at age 20; her death was reported by the family to have been a major psychological stressor for the patient. He did not receive antiseizure medications until after his second focal-to-bilateral tonic–clonic seizure at age 21, at which time levetiracetam was initiated but discontinued due to behavioral adverse effects. He was subsequently started on oxcarbazepine 600 mg twice daily, which effectively controlled the convulsive seizures, while his focal impaired-consciousness seizures persisted. At age 23, he had a routine EEG done which was reportedly within normal limits. The patient reported good mood and healthy sleep. He denied panic attacks, functional seizures, and any other history of psychological disorders.

According to his mother, the patient experienced two seizure types. One consisted of generalized convulsive events described as “full-body shaking,” consistent with focal to bilateral tonic–clonic seizures. The other consisted of a subjective sensation of falling accompanied by intense fear, loud vocalizations, and urgent pleas to be held tightly to prevent perceived imminent death. These seizures typically lasted approximately 2 min and were preceded by an aura of intense fear, lightheadedness, and palpitations. Postictal symptoms included coughing, confusion, and complete amnesia for the events.

**Figure 1:**
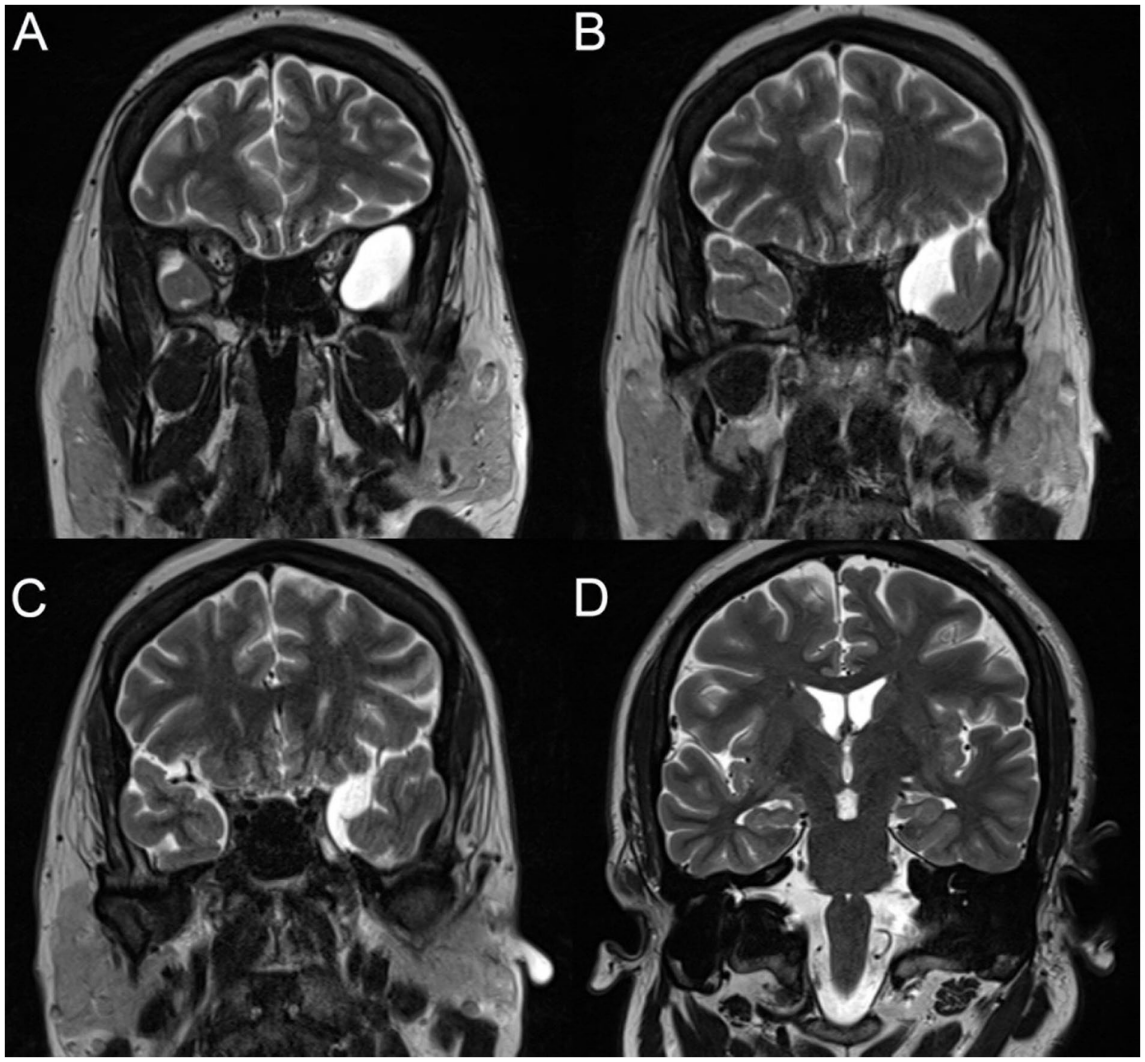
Successive T2-weighted coronal MRI of the brain showing the left temporal arachnoid cyst **(A–C)**. The hippocampi appeared within normal limits on a more posterior cut **(D)**.

Brain MRI demonstrated an arachnoid cyst in the left middle cranial fossa abutting the medial temporal lobe (Figure 1). Routine scalp EEG performed while the patient was taking oxcarbazepine was unremarkable. He was therefore admitted to the epilepsy monitoring unit for seizure characterization and optimization of medical management.

He was admitted to the Epilepsy Monitoring Unit (EMU) for a total of 5 days, and the detailed medication regimen is outlined in Table 1. At admission his oxcarbazepine level was 28 ug/mL. At discharge, he was started on zonisamide 150 mg twice a day and his oxcarbazepine dosage was increased to 900 mg twice a day.

**Table 1:** Antiseizure medication regimen during EMU admission.

Date	AM	PM
Day 1	Oxcarbazepine 600 mg	Oxcarbazepine 600 mg
Day 2	Oxcarbazepine 600 mg	Oxcarbazepine 600 mg
Day 3	No medication given	No medication given
Day 4	No medication given	Oxcarbazepine 900 mg, Zonisamide 100 mg
Day 5	Oxcarbazepine 900 mg, Zonisamide 150 mg	Discharged

During admission, four stereotypical seizures were recorded, two of which occurred during sleep. All seizures began with panicked, loud, and comprehensible verbalizations accompanied by intermittent lip smacking. The content of the ictal speech was consistent across events, with language selection varying according to the social context. When his mother was present, he addressed her in either Urdu or English; when speaking to medical staff, he used English exclusively. During each seizure, he called for help and repeatedly expressed fear that he was in danger and about to die, pleading to be held or hugged to prevent his death. He referenced his deceased sister and warned his mother that she would find his “corpse” if she did not hold him.

In two seizures, the patient described a subjective sensation of diffuse bodily shaking and possible redness. He asked his mother and staff to confirm whether visible shaking or redness was present, although neither was observed by witnesses. In three seizures, the onset of verbalization preceded the electrographic onset by approximately 5–10 s.

**Figure 2:**
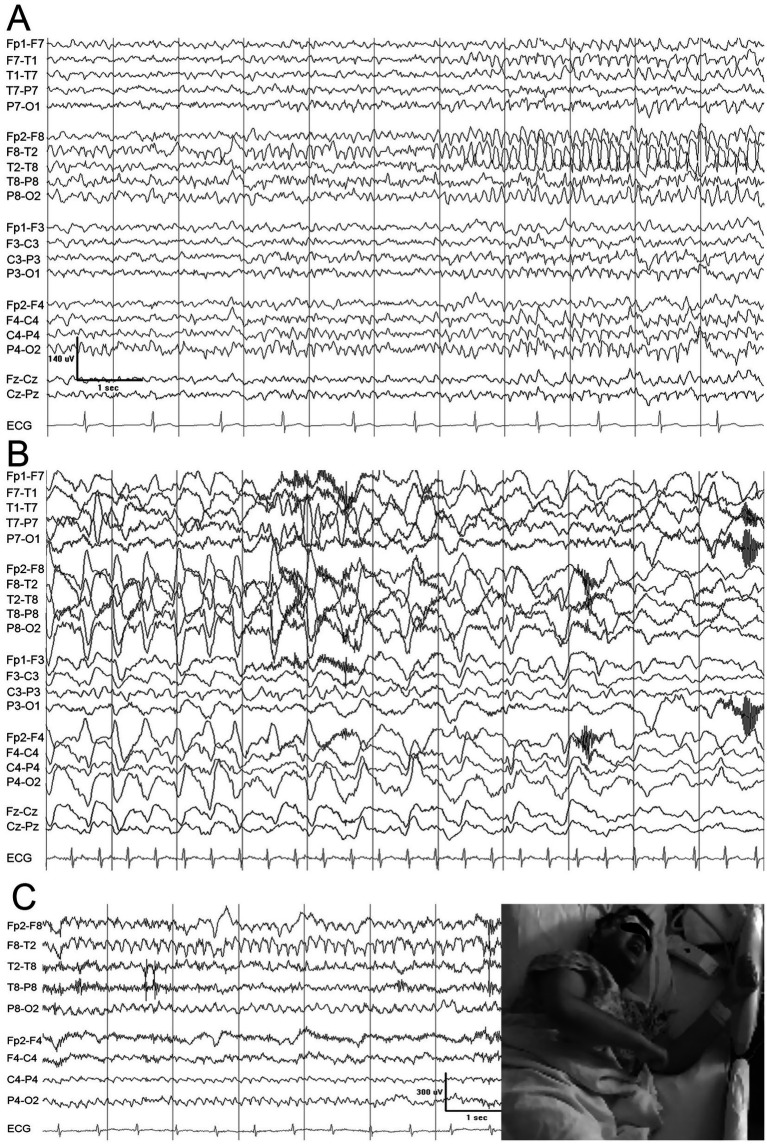
The onset of the ictal discharge **(A)** consists of an irregular, disorganized background over the right temporal derivations, which soon evolves into an organized, theta-range, spike discharge maximum over the anterior right temporal area (T2). Before the ictal discharge abates, it evolves into high voltage regular delta, maximum on the right **(B)**. The semiology did not differ between the seizure onset **(A)** and end **(B)**. Synchronous video-EEG showing the patient fearfully screaming “I’m going to die” with the right temporal ictal discharge organizing over the right temporal derivations **(C)**.

All seizures demonstrated a consistent electrographic pattern. Seizure onset was characterized by arciform 7-Hz theta activity localized to the right frontotemporal region, with maximal involvement at F8/T2 > T4 > T6. Activity subsequently spread to F10 and T10 and evolved into high-amplitude rhythmic 4-Hz notched delta activity with superimposed fast frequencies. The ictal rhythm then propagated to the right parasagittal region before transitioning to high-amplitude 3-Hz delta activity. Seizures terminated with generalized 2-Hz delta activity of increasing amplitude. Figure 2 shows representative samples from the video-EEG recording; detailed descriptions of the recorded seizures are provided below.

### Seizure 1

The patient was asleep at onset. Approximately 10 s before electrographic onset, he abruptly began speaking in English, shouting urgently for help (e.g., “Mom… somebody help me! I am in danger”). As oral automatisms emerged, his mother attempted to comfort him, speaking in Urdu.

He continued to vocalize loudly with fearful content, repeatedly expressing that he was about to die unless held, and warning his mother that she would find his body if she did not hold him. He referenced his deceased sister in urging his mother to respond (“Would you like to lose me like you lost your daughter? … hold me or I swear to God you’ll see your son’s dead body”).

### Seizure 2

The patient was asleep at onset. Approximately 38 s after electrographic onset, he began speaking in Urdu, addressing his mother by her first name and urgently asking her to come to him, tying his survival to her physical presence (e.g., pleading to be held in order to remain alive).

As his mother comforted him, he opened his eyes and hugged her. When a nurse entered the room, the patient switched to English, asking: “Can you just hold me?” Chewing automatisms were observed. He then stated: “I might get a seizure”.

### Seizure 3

This seizure began during an ongoing conversation with his mother, approximately 5 s before electrographic onset. With his hands clasped in a prayer-like posture, he stated in English: “Mom, I am feeling dizzy. I am about to get another seizure.” He then switched to Urdu, repeatedly asking his mother to hold him. When she declined, explaining that the medical team needed to observe him on camera, he became increasingly distressed and urgently called for help in English, stating that he was “fighting life and death” and feared imminent death. When a nurse entered the room, he asked whether visible shaking or redness could be observed. He was reassured that none was seen, but insisted that his entire body was shaking.

### Seizure 4

This seizure occurred while the patient was awake and speaking on the phone. Approximately 40 s before electrographic onset, he began blinking his right eye, followed by bilateral eye blinking. He then announced that he was about to have another seizure and asked for assistance. Oral automatisms were observed. He subsequently vocalized loudly with fearful content, repeatedly stating that he was about to die and urgently requesting to be held in order to remain alive. He again referenced the death of his sibling while pleading for physical contact and reassurance, insisting that his body was shaking despite the absence of observable motor activity.

When asked about his subjective experience during the seizures, the patient reported no recollection of the events. He expressed surprise upon being informed that he appeared frightened and repeatedly pleaded to be held. After listening to his ictal vocalizations on the recorded video, he displayed marked astonishment.

## Discussion

This case illustrates an unusual seizure semiology characterized by intense fear of death, loud and coherent vocalizations, contextually appropriate bilingual language use, accurate autobiographical references, and complete postictal amnesia, features that collectively imply engagement of a broad, integrative network. The patient consistently addressed his mother in Urdu or English while speaking exclusively in English to staff, demonstrating preserved pragmatic awareness of his social environment. Notably, he referenced his deceased sister during seizures despite complete amnesia afterward, highlighting a dissociation between access to autobiographical information during the seizure and subsequent consolidation failure.

Ictal fear is a recognized but relatively uncommon manifestation, most often linked to the amygdala and mesial temporal networks (2–5). In typical cases, fear is brief and nonverbal (6); extended, coherent speech with preserved syntax, pragmatic appropriateness, and autobiographical specificity is rare. The sustained verbalization of impending death in this patient resembles the case reported by Biraben et al., but differs in its linguistic richness and social contingency, suggesting concurrent engagement of limbic and higher-order associative networks (7).

Complete postictal amnesia with preserved behavioral responsiveness and language output aligns with “pure amnestic seizures,” thought to reflect transient disruption of mesial temporal memory encoding—particularly bilateral hippocampal structures—while sparing online retrieval and expression (8–10). This dissociation underscores the separation between real-time cognitive access and durable consolidation.

The phenomenology of this patient’s seizures shares partial overlap with neural systems engaged by mortality-related cognition (existential threat). Experimental work implicates a distributed network including the amygdala, anterior cingulate cortex (ACC), posterior cingulate cortex, caudate, and parietal association regions in self-referential and regulatory processing (1, 11). The right amygdala, in particular, appears to show heightened sensitivity to threat detection and emotional arousal during mortality-related cognition, even in the absence of conscious fear (1). The rostral ACC is similarly implicated—consistent with anticipatory anxiety, conflict monitoring, and processing of socially relevant threat—with several studies demonstrating greater activation to death-related cues than to other aversive stimuli (1). Of note, the ictal discharge was observed on the right in our subject, but a left hemispheric onset cannot be ruled out since he has a left temporal arachnoid cyst and exhibited a delay between clinical onset and ictal EEG changes. Scalp EEG has known limitations, particularly for deep or mesial temporal generators, and may preferentially capture propagated activity rather than the seizure onset. Early low-amplitude ictal discharges may remain below the detection threshold. Accordingly, we cannot confirm whether the arachnoid cyst is a mere incidental finding.

Epilepsy studies support overlapping circuitry for ictal fear and existential threat: amygdala activation can induce profound dread and autonomic changes; the insula contributes to interoception and subjective threat; ACC–amygdala interactions modulate fear regulation; and mesial temporal structures (including the hippocampus) can allow autobiographical content to surface during seizures. Subcortical modulators (thalamus, brainstem) may amplify autonomic sensations relevant to seizure-related fear and SUDEP risk (12). Conceptually, seizure-associated fear of death can be viewed as a pathological, temporally compressed overactivation of circuits that subserve existential threat in non-pathological conditions.

A motivational distinction offers a heuristic: existential threats preferentially recruit the Behavioral Inhibition System (BIS), centered on medial frontal/ACC circuits, promoting vigilance and sustained anxiety, whereas immediate physical threats more prominently engage fight–flight–freeze systems (13). Mortality salience is associated with prolonged self-referential processing and heightened vigilance (e.g., ACC activation, late positive potentials), unlike the typically brief affective surge of nonexistential threats (14, 15). This framework may help explain the unusually prolonged, narratively elaborated ictal fear observed here.

Individual differences modulate mortality-related neural responses: secure attachment, social-coalitional traits (e.g., leadership), and high self-esteem appear to buffer engagement of self-referential and parietal networks, whereas insecure attachment, low self-esteem, or pre-existing fear of death amplify such responses (16–18). While not directly measured in our patient, these factors may shape vulnerability to elaborated fear content during seizures.

In summary, convergent evidence from epilepsy and functional neuroimaging indicates that fear of death recruits overlapping—though not identical—substrates across physiological and pathological contexts. The amygdala, insula, mesial temporal regions, and ACC constitute a core network for both existential fear and ictal dread, with differences in context, temporal dynamics, and phenomenology. Seizures provide a unique, paroxysmal window into these systems.

Recognizing this overlap enriches interpretation of seizure semiology and cautions against misdiagnosis as primary psychiatric conditions, while highlighting how death-related threat processing is embedded in fundamental human cognition and emotion (19).

## Data Availability

The original contributions presented in the study are included in the article/supplementary material, further inquiries can be directed to the corresponding authors.
